# The effects of Salvia officinalis L. on granulosa cells and in vitro maturation of oocytes in mice

**Published:** 2017-10

**Authors:** Malihezaman Monsefi, Akram Nadi, Zeinab Alinejad

**Affiliations:** *Department of Biology, School of Sciences, Shiraz University, Shiraz, Iran.*

**Keywords:** Granulosa cell, In vitro maturation, Oocytes, Ovary, Salvia officinalis

## Abstract

**Background:**

*Salvia officinalis* *L.* has been used since ancient times but there are little data about effects of this herb on normal reproductive cells.

**Objective:**

To investigate the toxicity effects of *Salvia officinalis L.* on granulosa cells (GCs) and maturation of oocytes.

**Materials and Methods:**

GCs and oocytes were extracted from superovulated ovaries of immature mice. The cells were treated with concentrations of 10, 50, 100, 500, and 1000 μg/ml of *Salvia officinalis* hydroalcoholic extracts and compared with the control culture. Bioviability, chromatin condensation, estradiol and progesterone concentrations, lipid synthesis, apoptosis, and alkaline phosphatase activity of GCs were measured. In vitro maturation of oocytes by determination of different maturation stages of oocytes including germinal vesicle, germinal vesicle breaks down, and metaphase II were examined.

**Results:**

The results revealed that 500 and 1000 μg/ml concentrations of *Salvia officinalis* *L.* were toxic. The most of the GCs were in the early stages of apoptosis in 100 μg/ml treated culture and cell death happened with 500 μg/ml treatment. Progesterone concentration was reduced in 100 μg/ml and higher doses but estradiol concentration and alkaline phosphatase showed opposite effects. The lipid droplets content of GCs reduced significantly in all groups especially in 500 and 1000 μg/ml. Finally, oocyte’s nucleus and cytoplasm showed a high level of condensation, and meiosis rate reduced in all treated cultures.

**Conclusion:**

Our findings suggested that higher dose of *Salvia officinalis* hydroalcoholic extracts inhibits, oocyte maturation, GCs bioviability, proliferation, and secretion.

## Introduction

People may use herbal medicines as a complementary or alternative therapy to feel better or control their diseases. *Salvia officinalis L.* (sage), as an important plant of lamiaceae family, has been cultivated since ancient times. Sage tea has traditionally been used for the treatment of digestive and circulation disturbances, bronchitis, angina, depression, excessive sweating, skin diseases, and many other diseases (1-3). 

*Salvia officinalis L.* contains 1-2.8% essential oil and its main components include: α- and β-thujones (35-60%), flavonoids, phenolic acids (caffeic, chlorogenic, ellagic, ferulic, gallic, labiatic, and rosmarinic) (4, 5). *Salvia officinalis* is typically considered to have the highest amount of essential oil compared to other species of Salvia (6). Flavones and flavonoids of *Salvia officinalis* are known as phytoestrogens. Ursolic acid (2.1%) effectively inhibits angiogenesis and invasion of tumor cells and metastasis (7). Sage has antioxidant activity, and has introduced as an anti-carcinogenic herb but there are few data about probable side effects on reproductive system (8, 9).

According to our previous data, epithelial cells of mammary gland showed estrogenic activity, higher growth and cell division when treated with *Salvia officinalis* extract (SHE) in adult female rats (10). Additionally, it was found that the same dose of this extract decreased significantly the growth and cell division in mammary glandular cells when exposed to estrogen-like substance, dimethyl1,7[a]bens-antheracene (DMBA) for breast cancer induction in adult female rats (unpublished data). Therefore this herb has showed two different effects due to estrogen concentration. 

An ovary, as the source of ova, possesses two primary steroidogenic cell types of the theca cells and the granulosa cells (GCs). It was shown that phytoestrogens of *Salvia officinal L*. can interfere with steroidogenesis activity of ovary. We did not find any reports about effects of this herb on ovarian cells which are very important for reproduction. GCs culture and in vitro maturation (IVM) are two major tools that provide excellent systems for studying the effects of drugs and toxic compounds on the reproductive system. Moreover, the effects of different foods and nutrition such as high-fat diet on oocyte quality and fertilization rate have been evaluated by these methods (11). 

The effects of *Salvia officinalis L.* hydroalcoholic extract on mouse ovarian GCs and IVM of oocytes were taken into consideration.

## Materials and methods

**Animal**

This study was performed at Animal Cellular-Developmental Biology Laboratory, Biology Department, College of Sciences, Shiraz University between March 2014 and September 2015. Immature Balb/c female mice 25-35 days (n=20) weighing 13-18 gr were purchased from the Animal House of Shiraz University of Medical Sciences, Shiraz, Iran. The mice were adapted to the laboratory conditions for 2 wk prior to the experiments. Animals were kept at a controlled temperature (22-24^o^C) and a 12 hr light/dark cycle (lights on from 6:00 until 18:00); they had free access to food and tap water. 

**Preparation of extract**

*Salvia officinalis L.* leaves were obtained from Zardband Pharmaceuticals Company, Tehran, Iran. It was identified by a botanist in the Herbarium of the Fars Natural Resources Research Center, Shiraz, Iran. A voucher specimen was also preserved for reference with the serial number 1128. Sage leaves were powdered, and 100 g of powder was percolated with 800 ml of 70% ethanol for three days. Subsequently, the mixture was filtered and concentrated under reduced pressure using a rotary evaporator and vacuumed desiccator. The yield (w/w) of SHE was 17% (g/g).

**Ovarian granulosa cells culture**

Twelve immature female mice were stimulated by an intra-peritoneal (i.p.) injection of 7 IU pregnant mare serum gonadotropin (Hypra, Spain). The animals were sacrificed 48 hr later by cervical dislocation and the ovaries were removed and placed into Petri dish containing Dulbecco’s Modified Eagle’s Medium (DMEM)/F12 (Gibco, USA) supplemented with 20% fetal bovine serum (FBS) (Gibco, USA), 1% penicillin-streptomycin (Sigma-Aldrich, USA) and 0.1% bovine serum albumin (BSA) (Gibco, USA). Ovaries were subjected to puncture with 25-gauge needle and follicles were separated and transferred to the other Petri dish. Follicles were punctured again to GCs were released then GCs were aspirated aseptically in new media. They were centrifuged (500 g for 5 min), then resuspended in cultivation medium and seeded. 

Cells number and viability were estimated using a hemocytometer under a light microscope after vital staining with trypan blue (Sigma-Aldrich, USA). Number of 5×10^5^ cells were grown and maintained in 0.5 ml DMEM/F12 containing 20% FBS, and 1% penicillin-streptomycin and 0.1% BSA per each well of 24 well plate. SHE were added to the culture media at concentrations of 0 (Control culture), 10, 50,100, 500, 1000 and 10000 mg/ml after 24 hr. The osmolality of the extract containing media was adjusted to 300-320 miliosmol with an osmometer (Gonotec GmbH, Rinteln, Germany). All cells were incubated at 37°C with 5% CO_2_ for two days. Each concentration was repeated in 3 wells of 24-well plate (n=3) separately. 

**Cell viability assay**

Cell viability was assessed by neutral red (0.05%) (Merck, Germany) for 2 hr at 37^o^C, Then the cells were fixed in formal Ca 1 min at RT and washed 2 min in saline. Subsequently, 1 ml alcohol acid was added and the mixture was incubated 2 hr at RT. The optic density of the eluted neutral red in alcohol acid was measured at 540 nm wavelength by spectrophotometer (Shimadzu UV-120-01, Kyoto, Japan). 

**Estradiol and progesterone measurements**

A number of 5×10^5^ cells were cultured or grown in 0.5 ml DMEM/F12 containing 20% FBS, and 1% penicillin-streptomycin and 0.1% BSA per each well of 24-well plate. Furthermore, SHE was added to the culture media at concentrations of 0, 10, 50,100, 500 and 1000 mg/ml after 24 hr. GCs were incubated at 37^o^C with 5% CO_2_ for 48 hr. Each concentration was repeated in 3 wells of 24 well plate (n=3) separately. For hormone preservation, the medium was not exchanged during this period. 

The supernatant were collected from each well and centrifuged. Estradiol concentration in different SHE dosage was measured using radioimmunoassay (RIA) method by estradiol kit (Diasource, distributor Aria Pharmed Producing and Trading, Tehran, Iran) and progesterone concentration was measured using immunoradiometric assays (IRMA) by progesterone kit (Diasource, distributor Aria Pharmed Producing and Trading, Tehran, Iran) in Department of Hormonal assay, Research Center of Namazi Hospital, Shiraz, Iran. 

**Alkaline phosphatase (ALP) activity assessment**

GCs were cultured with the same method for hormonal measurement. ALP activities of GCs supernatants were examined by ALP kit (Kimia Pajouhan, Tehran, Iran). ALP converted colourless paranitrophenyl phosphate as a substrate to yellow paranitrophenol and phosphate. Color intensity has a direct proportion of enzyme activity that was measured by the spectrophotometer (Jenway, Staffordshire, England) at 405 nm wavelength.

The Lambert and Beer’s law was used to calculate ALP activity. According to this law, when monochromatic light passes through a coloured solution, the light absorbance (A) depends on the pigment concentration (C) of the solution and the thickness of the tube (L) that light passes through it or A=KCL (K: coefficient of absorption). The tube diameter is considered 1 cm, so A=KC and C=A/K. Light absorption was measured at different times, ΔC=ΔA/K. 

**Chromatin condensation assay**

GCs were cultured on 12 mm round sterile coverslips that put bottom per each well of 24-well plate. Cells were treated with different doses of sage hydro-alcoholic extract for 48 hr. 4T1 cells were fixed in 3% glutaraldehyde in PBS 0.2 M 30 min then stained with 5% aniline blue (Acros Organics, USA) in 4% acetic acid 10 min at pH=3.5. The coverslips were removed and put on the slide and examined under light microscope, and then their photographs were taken by a digital camera (Nikon, Japan). Condensed chromatin was stained with dark blue (12). The light intensity of 100 nuclei in each concentration was analyzed by Image Java software. This software represents light intensity as a number in the range between 0 to 255 which 0 represents the absolute black and 255 represents the absolute white. Cells with more condensed chromatin look darker and acquire lower score in the Image Java calculation. 

**Acridine orange/ethidium bromide (AO/EB) staining**

AO/EB staining is used for evaluation of nuclear morphology in apoptotic cells (13). After the treatment period, GCs were harvested and rinsed with PBS. The pellets were resuspended in AO/EB solution including 5 μL of AO (Merck, Germany) and 5 μL of EB (SinaClon, Iran). After 10 min, the cells were put on the slide and observed using a fluorescence microscope (Nikon Eclipse-E600) and photographs were taken at ×10 magnification using a digital camera (Nikon, Japan). Acridine orange is a vital dye and stains both live and dead cells. Ethidium bromide stains only cells that have lost membrane integrity. Live cells will appear uniformly green. 

Early apoptotic cells stain green and contain bright green dots in the nuclei as a consequence of chromatin condensation and nuclear fragmentation. Late apoptotic cells also incorporate ethidium bromide and therefore stain orange, but, in contrast to necrotic cells, the late apoptotic cells show condensed and often fragmented nuclei. Necrotic cells stain orange but have a nuclear morphology resembling that of viable cells, with no condensed chromatin.

**Oil red o staining of GCs**

GCs secrete steroid hormones, and they have some lipid droplets in their cytoplasm. Oil red o is a lysochrome (fat-soluble dye) used for staining of neutral triglycerides and lipids (14). GCs were cultured in different SHE concentrations in 24-well plate. The bottom of each well was covered by sterile round coverslip previously. After 72 hr, the culture medium was discarded, and cells were fixed with 4% formalin contained 1% calcium chloride for 15min and washed with 70% ethanol. 

Then 500 μl oil red o (Sigma-Aldrich, USA) solution (250 mg oil red o were dissolved in 5 ml of 99% isopropanol then 3 parts of this solution were added to 2 parts of dH_2_O) were added to each well. After 15 min dye solution was discarded and cells washed with 70% ethanol and dH_2_O respectively. The coverslip was removed and placed on a glass slide and observed by light microscope. GCs were then evaluated as three groups of low, medium and high containing of lipid droplet. Then 100 cells were counted and the percent of each group were recorded in different SHE concentration. 

**Collection of oocytes**

Oocytes were obtained from 25-35 days old Blab/C female mice. The mice were stimulated by an i.p. injection of 17 IU pregnant mare serum gonadotropin (Hypra, Spain) and 17 IU of human chronic gonadotropin (HCG) (LG Life Sciences, South Korea) after 48 hr. The animals were sacrificed 16 hr later by cervical dislocation and the ovaries were removed into minimum essential medium-alpha (MEM-Alpha) (Gibco, USA) supplemented with 10% fetal bovine serum (FBS) (Gibco, USA), 1% penicillin-streptomycin (Sigma-Aldrich, USA).

Oocytes of ovarian follicle were aseptically harvested by aspiration from follicles with a 22 and 31-gauge sterile needles and released in the medium under a stereomicroscope. Cumulus cells were removed by repeated pipetting and the oocytes were collected for IVM (11). A total of 800 denuded oocytes were obtained from 16 ovaries of 8 mice (4 repeated examinations) and they were used for IVM. Besides, the average number of collected oocytes was 50 per ovary.

**In vitro maturation**

The collected oocytes in each examination were randomly divided into control and seven experimental groups. Each group was placed in 35 μl micro drops of maturation medium that consisted of MEM-Alpha supplemented with 10% FBS and 1% penicillin-streptomycin over laid with embryo tested light mineral oil and incubated for 24 hr in a humidified atmosphere of 5% CO_2_ at 37^o^C. In experimental groups, SHE at concentrations of 5, 10, 50, 100, 250, 500, and 1000 mg/ml were added to the culture media. The osmolality of the extract containing media was adjusted to 300-320 miliosmol with an osmometer (Gonotec GmbH, Rinteln, Germany). 

After 24 hr incubation, oocytes were observed by a stereomicroscope. Morphological changes in the nucleus or extrusion of condensed oocytes and the first polar body (MΙΙ) were used at the criterion for nuclear maturation of germinal vesicle (GV) stage oocytes in each SHE concentration and then their media were exchanged. After 48 hr, oocytes in the different group were observed again and the stages of each oocyte were recorded.

**Oocytes staining**

After 48 hr, oocyte’s viability was estimated after vital staining with trypan blue (Sigma-Aldrich, USA). In live oocytes with intact cell membranes, trypan blue was not absorbed and consequently, it was not coloured. However, this dye traversed through a membrane in a dead cell (enters cytoplasm) and was shown as a distinct blue colour under a microscope. The structures of the chromosomes in different stages of oocytes in each concentration were determined by aceto-orcein staining. Oocytes were fixed in an acetomethanol (acetic acid: methanol, 1:3) solution for 24 hr at 4^o^C. Fixed oocytes were transferred onto a microscope slide, and were covered by coverslips. Oocytes were incubated 10 µl of aceto-orcein solution (1% orcein, 45% acetic acid) for 2-3 min under a coverslip and then the structure of the chromosomes was analyzed.

**Ethical consideration**

The animal experiments were also approved by the Institutional Animal Ethics and Health Committee of the Biology Department of Shiraz University (No, 9130633), and were performed according to the principles of the care and use of laboratory animals established by the NIH.

**Statistical analysis**

The gathered data were analyzed via One-Way ANOVA, followed by the Tukey and Scheffe tests. Statistical analyses were done with Statistical Package for the Social Sciences, (SPSS, version 17.5, SPSS Inc, Chicago, Illinois, USA). P<0.05 was considered as the statistically significant difference. All data have normal distribution.

## Results

**Figure 1:**
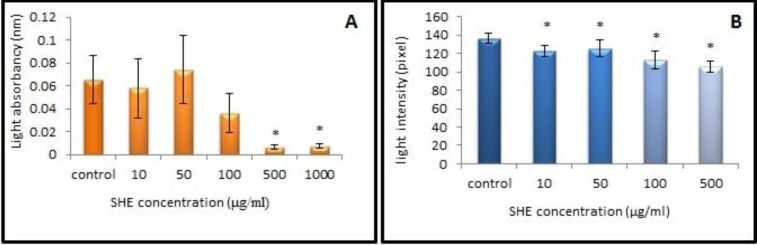
The effects of the different doses of *Salvia officinalis* on granulosa cells viability (A) and chromatin condensation (B) after neutral red and aniline blue staining respectively.

**Figure 2:**
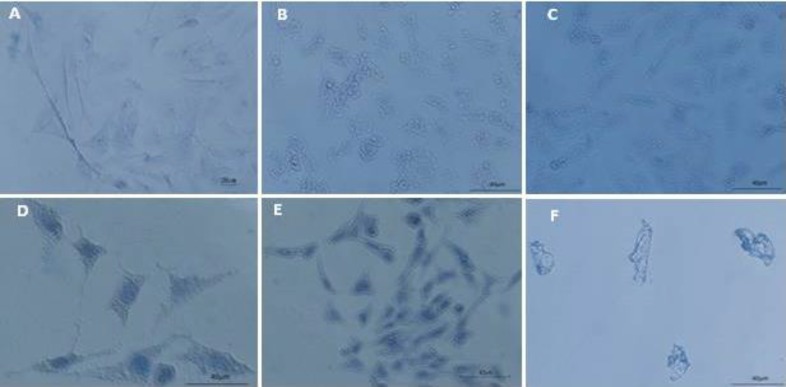
The effects of the different doses of *Salvia officinalis* on chromatin condensation of granulosa cells, aniline blue staining, scale bar=40µm. A) Control culture, B) 10 μg/ml, C) 50 μg/ml, D) 100 μg/ml, E) 500 μg/ml and F) 1000 μg/ml of *Salvia officinalis* extract treated culture. Dark and condensation of nuclear chromatin were noted. There are some apoptotic cells in 1000 μg/ml of *Salvia officinalis* extract treated culture

**Figure 3:**
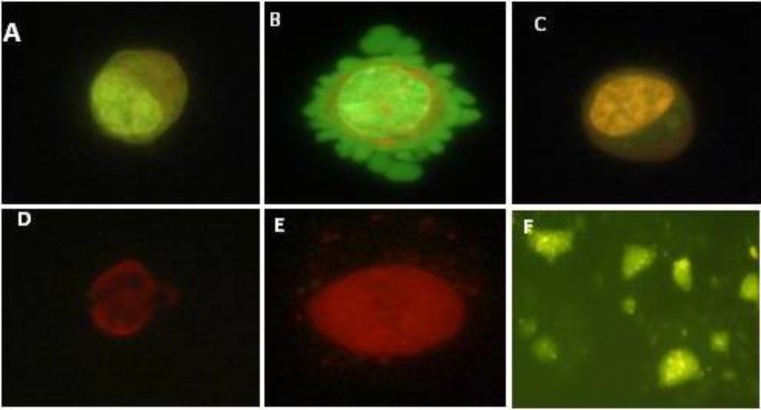
Ovarian granulosa cells under acridine orange/ethidium bromide (AO/EB) staining, A) Viable cells with a green nucleus, B) chromatin condensation and the formation of blebs on the cell surface associated with apoptosis assayed by acridine orange and ethidium bromide staining, C) cell with a yellow nucleus in the primitive apoptosis, D) cell with a red dried up nucleus in the apoptosis, E) necrotic cell with a red usual nucleus, F) The cell splintered in 1000μg/ml concentration

Cells viability assays showed that 10, 50 and 100 μg/ml of SHE concentrations did not affect GCs and they were similar to the control culture. However, at 500 and 1000 μg/ml of SHE concentrations, viable GCs decreased significantly (p= 0.02, p=0.01 respectively) (Figure 1). GCs in the control culture showed round euchromatin nuclei in the centre of their cytoplasm using aniline blue staining. SHE concentrations of 10 and 50 μg/ml condensed chromatin 1.1 fold when compared to the control culture (p<0.001). Higher concentrations of SHE (100 and 500 μg/ml) showed more 1.3 fold condensed chromatin than to the control GCs (Figures 2, 3) (p<0.001). In addition, chromatin condensation showed a significant statistical difference when the different doses compared together (p<0.001).

**Figure 4:**
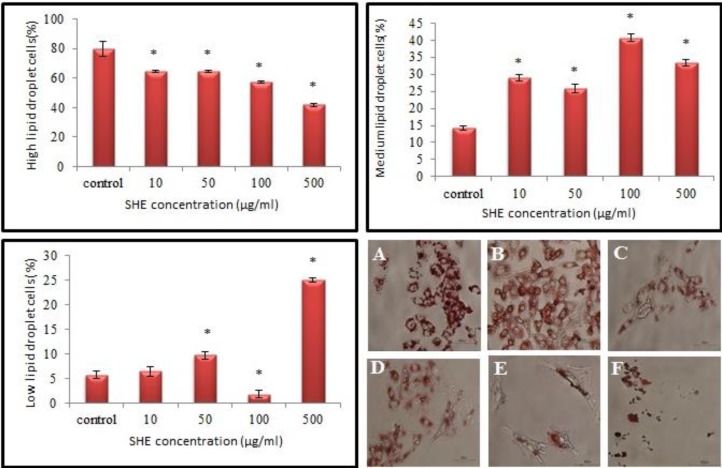
The effects of the different doses of *Salvia officinalis* on high, medium and low contents of lipid droplet of granulosa cells cultures, oil red staining, scale bar=40µm. A) Control culture, B) 10 μg/ml, C) 50 μg/ml, D) 100 μg/ml, E) 500 μg/ml and F) 1000 μg/ml of *Salvia officinalis* extract treated culture.

GCs of the control culture showed a dark green colour using AO/EB staining (Figure 4A). GCs of 10 μg/ml concentration of SHE was represented as light green with low red granule in their cytoplasm (Figure 4B). GCs of 50 μg/ml concentration of SHE was similar to 10 μg/ml but they showed more cytoplasmic granule and a few cell membrane blebbing. GCs in 100 SHE concentration cultures were mainly in the early stage of apoptosis and showed yellow nuclei, membrane blebbing and cytoplasm granulation (Figure 4C). Dead cells were shown by a red colour in 500 and 100 μg/ml of SHE (Figure 4D, 4E). The percentage of live cells decreased significantly at higher concentrations of the SHE extract, with opposite results for dead cells.

**Figure 5:**
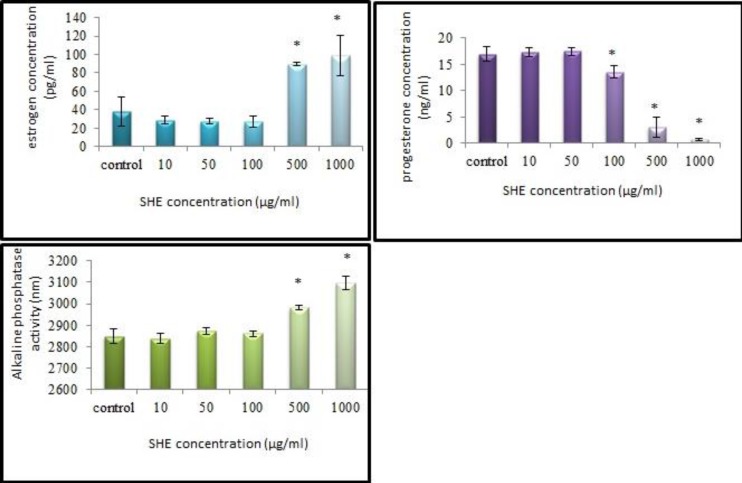
The effects of the different doses of *Salvia officinalis* extract on estradiol and progesterone concentrations and alkaline phosphatase activity of granulose cell culture.

GCs of the control culture showed the highest percentage of high lipid droplet cells, low percent of medium lipid droplet cells and the lowest percent of low lipid droplet cells. The cytoplasm of GCs treated with sage extract showed the lower percentage of high content of lipid droplet but a higher percentage of medium and low contents of lipid droplets compared to the control GCs culture significantly. SHE concentrations of 100 and 500 μg/ml revealed the highest percent of medium and low lipid droplet cells respectively (p<0.001) (Figure 5). GCs treated with 1000 μg/ml of SHE concentration appeared as dead and shrink cells (Figure 5).

**Figure 6:**
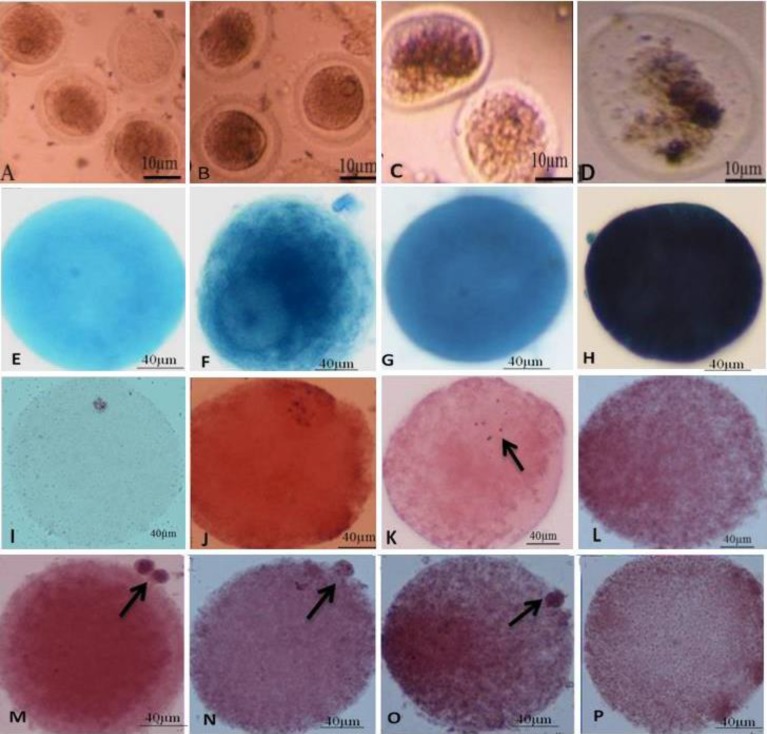
The effects of different doses of *Salvia officinalis* extract on different stages of oocytes in culture. Bioviabilityof denuded and unstained oocytes in A: control culture, B: 50 μg/ml, C: 250 μg/ml and D: 1000 μg/ml of *Salvia officinalis* extract cultures. Oocyte viability examination after trypan blue staining in E: control culture, F: 10 μg/ml, G: 100 μg/ml and H: 1000 μg/ml of *Salvia officinalis *extract cultures. The structures of the chromosomes in GVBD stage after aceto-orcein staining in I: control culture, J: 5 μg/ml, K: 250 μg/ml and L: 500 μg/ml of *Salvia officinalis *extract cultures. The polar body formation in MII stage after aceto-orcein staining in M: control culture, N: 5 μg/ml, O: 50 μg/ml and P: 1000 μg/ml of *Salvia officinalis* extract cultures

Estradiol concentration of GCs control culture and SHE concentrations of 5, 10, 50 and 100 μg/ml were similar but it increased in 500 and 1000 SHE concentrations significantly (p<0.001) compared to the control and the other SHE concentrations cultures (Figure 6). However, progesterone concentration showed the reverse results since its concentration in GCs control culture and SHE concentrations of 5, 10, 50 and 100 μg/ml were low but it decreased severely in 500 and 1000 SHE concentrations (p<0.001) (Figure 6). ALP activity of GCs control culture and SHE concentrations of 5, 10, 50, and 100 μg/ml were similar. This enzyme activity increased in 500 and 1000 μg/ml SHE concentrations significantly (p<0.001) compared to the control and the other SHE concentrations cultures (Figure 7).

Table I represents the number of GV, condensed, GVBD and MII stages oocytes. The number of GV stage oocytes decreased significantly in SHE treated cultures after 24 hr (p<0.017, p<0.002, p<0.001, p<0.001 and p<0.01); but it was prominent in higher doses of extract such as 500 and 1000 μg/ml compared to the control culture (p<0.001). The number of oocytes did not show any difference in SHE treated cultures after 48 hr except in high doses of 500 and 1000 μg/ml concentrations of extract. The oocytes with condensed cytoplasm and nuclei increased in all SHE treated cultures after 24 and 48 hr (Figures 6 A-D) (p<0.001to p<0.002). The number of GVBD and MII oocytes decreased in all SHE treated culture so that there were no oocytes in these stages in 500 and 1000 μg/ml treated cultures compared to the control culture (p<0.001). The stages of oocytes of different doses of SHE showed significant differences when they examined using between group analysis (p-value between 0.03 to 0.009). The number of GV stage oocytes decreased but condensed oocytes increased in all doses significantly after 48 hr when they compared to the cultured oocytes after 24 hr (p<0.001) (Table I). The trypan blue and aceto-orcein staining showed that the breakdown of nuclear membrane and meiosis II division starting were reduced or stopped in a higher dose of SHE treated cultures (Figures 6 E-P).

**Table I:** The effects of different doses of *Salvia officinalis* extract on number of different stages of GV, GVBD and MII (metaphase II) after 24 and 48 h cultures

***Salvia officinalis*** ** extract (μg/ml)**	**Number of GV stage oocytes**		**Number of condensed oocytes**		**Number of GVBD stage oocytes**	**Number of MII stage oocytes**
	**24 hr**	**48 hr**	**24 hr**	**48 hr**		
0 (control)	38.8 ± 0.9	6.0 ± 0.8	1.3 ± 0.9	2.3 ± 0.5	24.3 ± 2.9	6.5 ± 1.9
5	35.3 ± 0.9*	7.0 ± 1.4	4.8 ± 0.9*	7.8 ± 0.9*	16.5 ± 1.2*	4.0 ± 0.8*
10	34.5 ± 1.7*	8.3 ± 2.6	5.5 ± 1.7*	10.3 ± 1.2*	13.8 ± 1.2*	2.5 ± 0.5*
50	32.0 ± 0.8*†	8.8 ± 0.9	8.0 ± 0.8*†	10.3 ± 0.5*	11.3 ± 1.5*†	1.8 ± 0.5*†
100	31.5 ± 1.3*†¶	7.8 ± 0.9	8.5 ± 1.2*†¶	12.8 ± 2.0*†	9.5 ± 1.2*†¶	1.5 ± 0.5*†
250	29.8 ± 0.5*†¶	6.0 ± 0.8	10.3 ± 0.5*†¶	17.3 ± 2.0*†¶§†	6.0 ± 0.5*†¶§†	0.0 ± 0.0*†¶
500	27.3 ± 2.0*†¶§†	5.5 ± 1.2§	12.8 ± 2.0*†¶§†	21.5 ± 1.2*†¶§†!	0.0 ± 0.0*†¶§†	0.0 ± 0.0*†¶
1000	24.0 ± 1.4*†¶§†!Ϊ	1.3 ± 1.5*†¶§†!Ϊ	16.0 ± 1.4*†¶§†!Ϊ	22.8 ± 2.6*†¶§†!	0.0 ± 0.0*†¶§†	0.0 ± 0.0*†¶

All data were presented as mean±SD

GV: Germinal vesicle

GVBD: germinal vesicle break down

MII: Metaphase II

* Significantly different from 0 μg/ml (p<0.05) using One-Way ANOVA test

† Significantly different from 5 µg/ml (p<0.05)

¶ Significantly different from 10 µg/ml (p<0.05)

§ Significantly different from 50µg/ml (p<0.05)

† Significantly different from 100 µg/ml (p<0.05)

! Significantly different from 250 µg/ml (p<0.05)

Ϊ Significantly different from 500 µg/ml (p<0.05)

*Significantly different from 0 μg/ml (P<0.05)

*Significantly different from 0 μg/ml (P<0.05)

## Discussion

Viability test revealed that neutral red absorption of GCs decreased in higher doses of SHE treated cultures but their growth in lower doses was similar to the control culture. Aniline blue reacts with chromatin and its more intense reaction reveals condensed nuclei in all doses of SHE treated cultures. It, however, showed a higher reaction in dose-dependent manner. There is an association between the viability reduction of GCs treated with SHE and degree of their chromatin condensation.

Chromatin condensation is one of the distinct morphological and biochemical changes occurring during apoptosis (15). Acridine orange, as a fluorescent dye, binds to double-strand DNA and produces a green colour, but ethidium bromide binds to the fragmented DNA of necrotic or apoptotic cells and produces yellow to red colours. In addition, increase in the concentration of SHE led to decrease of viable (green) cells, whereas an increase in the number of early apoptotic cells (yellow) and late apoptotic cells (orange). The results of both aniline blue and acridine orange/ethidium bromide staining confirmed cell viability decrease of GCs treated with SHE.

*Salvia officinalis* includes phytoestrogens compounds such as flavonoids (luteolin, apigenin, quercetin-glycosides) (16). Phytoestrogens are nonsteroidal components attach to alpha and beta estrogen receptors (ER), similar to natural estrogens, and induce biological effects (17). The phytoestrogen toxicity on GCs in high doses has been reported (18-20). 

Resveratrol, a natural phenol produced by sage, has been demonstrated to inhibit the proliferation of cells and has been shown to alter the cell cycle and induce apoptosis by interfering with the estrogen receptor (ER)-dependent phosphoinositide 3-kinase pathway. Resveratrol inhibits both NF-κB, a regulator of Bcl-2 expression, and calpain protease activity, a regulator of NF-κB (21). Quercetin and resveratrol increased Bax expression (22). Rosmarinic acid induces apoptosis in both cell lines of human colon carcinoma-derived HCT15 and CO115 (23). Our new research showed *Salvia officinalis L.* induces apoptosis in mammary carcinoma cells through alteration of Bax to Bcl-2 ratio (24).

Moreover, the total lipid droplets amounts of GCs reduced significantly in all groups especially in 500 and 1000 μg/ml concentrations. Lipid droplets might play an important role in hormones production, but the amount of lipid clearly does not always reflect hormone secretion (25). 

The progesterone and estradiol concentrations did not change in lower doses of SHE treated cultures but progesterone concentration decreased in higher dose whereas estradiol increased significantly that could be due to phytoestrogens of this herb. Phytoestrogens show either agonistic or antagonistic effects based on the estrogen level and estrogenic receptors saturation (26). Therefore the progesterone and estradiol concentrations decreased in dose-dependent manner of SHE treated culture. The most amount of estradiol in 500 and 1000 μg/ml may be due to phytoestrogen components of sage extract that interfere this hormone measurement. Some phytoestrogens such as quercetin, daidzein and genistein have been reported the similar results on steroidogenesis (18-20, 27).

The study also clearly showed that ALP activity was controlled by ovarian hormones (28). Estrogen along with progesterone increased the ALP activity and the endometrial thickness (29). Estradiol is able to modify progesterone action in a dose-dependent manner. Low doses of estradiol potentiate the progesterone effect, whereas its higher concentrations almost completely block progestational response. Progesterone in the mammalian uterus is regulated by its cytosolic receptor concentrations and uteroglobin, a progesterone-stimulated uterine protein (30). The most of the phytoestrogens and industrial chemicals behaved as estrogen on stimulation of ALP activity (31). 

Our data also revealed that IVM of oocytes after SHE treatment showed similar results to GCs cultures. Early oocytes classified as immature or GV stage. GVBD stage indicates a resumption of meiosis and the extrusion of the first polar body indicates completion of the first meiotic division in oocytes. *Salvia offisinalis* decreased the oocytes maturation stages in dose-dependent manner. The number of GV stage oocytes reduced with increasing dose of sage extract and oocytes led to condensed nucleus and cytoplasm. The nuclear breakdown and first polar releasing reduced significantly too. These changes were highly considerable after 48 hr from culture than to the 24 hr. It may be due to the phytoestrogen and antioxidant components of this herb that showed similar effects on GCs cultures. A low dose of genistein and daidzein and their metabolites did not affect oocyte maturation but its high dose caused oocytes apoptosis (32, 33). 

A low dose of quercetin as an antioxidant improved the in vitro development of porcine oocytes by decreasing reactive oxygen species levels but in higher dose showed the toxic effect and inhibited oocytes growth (34, 35). A lower dose of resveratrol protected mouse oocytes from methylglyoxal that induced oxidative damage and inhibited oocytes in GV stage (36). Administration of aqueous extract of a mixture of Medicago sativa and *Salvia officinalis* on female mice have revealed a significant increase in luteinizing hormone and estradiol while decrease of follicle- stimulating hormone level. Also increase the number of ovarian follicles and corpora lutea, endometrial glands diameter and uterine epithelial cells height have reported. The authors concluded that obtained results may be related to the phytoestrogen constituents of both plants (37).

## Conclusion

*Salvia officinalis* *L.* hydroalcoholic extract showed different behaviours on GCs growth, function, and oocytes maturation based on its administration dose. A low dose of this extract did not show any disorder to GCs and oocytes culture, but its high dose was toxic and inhibited GCs and oocytes bioviability and proliferation and induced apoptosis. It also implied that higher doses of this herb have side effects on fertility.
